# Modulating the Electronic Structure and Global Reactivity of Nitrogen/Boron Co-Doped Graphene Oxide: A Density Functional Theory Study for Enhanced Gas Sensing Applications

**DOI:** 10.3390/molecules31142456

**Published:** 2026-07-14

**Authors:** Awad M. Bakry, Lamiaa S. El-Sherif, Hegazy Rezk, Safwat Hassaballa, Hanan Elhaes, Medhat A. Ibrahim

**Affiliations:** 1 Department of Physics, College of Science and Humanities in Al-Kharj, Prince Sattam bin Abdulaziz University, Al-Kharj 11942, Saudi Arabia; 2 Department of Electrical Engineering, College of Engineering in Wadi Alddawasir, Prince Sattam bin Abdulaziz University, Al-Kharj 11942, Saudi Arabia; 3 Physics Department, Faculty of Science, Islamic University of Madinah, Almadinah Al-Munawarah 42351, Saudi Arabia; 4 Physics Department, Faculty of Women for Arts, Science and Education, Ain Shams University, Cairo 11757, Egypt; 5 Spectroscopy Department, National Research Centre, 33 El-Bohouth St., Dokki, Giza 12622, Egypt; 6 Center for Converging Sciences and Emerging Technologies (CoSET), Benha National University (BNU), Al Obour 13518, Egypt; 7 Molecular Modeling and Spectroscopy Laboratory, Centre of Excellence for Advanced Science, National Research Centre, 33 El-Bohouth St., Dokki, Giza 12622, Egypt

**Keywords:** graphene oxide (GrO), density functional theory (DFT), N/B co-doping, gas sensing, global reactivity descriptors and electronic structure

## Abstract

Density Functional Theory (DFT) calculations were applied at the B3LYP/6-311G+(d,p) level to examine how nitrogen (N) and boron (B) and combined nitrogen/boron doping (N/B) affected the electronic properties and chemical behavior of graphene oxide (GrO). The work aimed to measure how global reactivity descriptors, including ionization potential, chemical hardness, and electrophilicity, changed when dopants entered the system while evaluating their prediction accuracy for gas sensing performance against NH_3_ and H_2_O and CO_2_. The results show that undoped GrO exhibits a HOMO/LUMO gap value of 2.9059 eV while the introduction of dopants increases reactivity through gap reduction because N-doping decreased the gap to 1.3622 eV, B-doping reduced it to 1.3388 eV, and co-doping (GrO-NB) led to a gap of 1.9897 eV. The TDM analysis and the gas interaction energy gap results show that GrO-NB-H_2_O exhibits the strongest interaction which results in chemical reactivity through its lowest ΔE of 1.9565 eV, establishing itself as a highly sensitive water vapor sensor when compared with NH_3_ and CO_2_. With adsorption energies of −0.1986, −0.1742, and −0.0735 eV for NH_3_, H_2_O, and CO_2_, respectively, the N/B co-doped graphene oxide demonstrated favorable and reversible physisorption, underscoring its potential for gas sensing applications. The results offer an essential understanding of how N/B co-doping influences the electronic and adsorption characteristics of graphene oxide, thereby supporting its potential use in graphene-based sensing technologies.

## 1. Introduction

Graphene oxide (GrO) is recognized as the oxidized form of graphene, essentially a single-atomic-layer material derived from graphite that has been chemically treated to introduce many functional groups [1,2,3]. The presence of oxygen groups in GrO fundamentally transforms its properties compared with pure graphene, making it a highly versatile and cost-effective material for a wide array of applications in nanotechnology and material science. These oxygen functionalities enable superior dispersibility and reactivity and the ability to form advanced composites, which are critical for modern technological innovations [4,5,6]. The oxidation process disrupts the perfect sp^2^ bonding of pristine graphene by attaching oxygen-containing functional groups in a semi-random fashion across both the basal plane and edges. This leads to the formation of oxidized and non-oxidized regions, localization of π-electrons, band gap opening, and significant changes in the material’s electronic, mechanical, and chemical properties [7,8].

Atoms are incorporated into the graphene oxide lattice primarily through oxidation, which introduces oxygen-containing functional groups onto the carbon lattice, and through substitutional doping, where foreign atoms like silicon can replace carbon atoms, causing local structural distortions [9]. These modifications significantly alter the chemical and physical features of graphene oxide in comparison with pristine graphene. The modifications that convert pristine graphene to graphene oxide introduce oxygen-containing groups, which drastically alter its chemical reactivity, solubility, mechanical strength, and electrical and thermal conductivity, and open new application possibilities, albeit often at the expense of some of graphene’s exceptional intrinsic properties [10,11,12,13].

Nitrogen (N) and boron (B) are doped into graphene lattices, replacing carbon atoms. This process is widely used to modify both the physical and chemical features of graphene throughout enhancing the electronic properties [14,15].

Under high-temperature treatment or other synthesis methods, N and B atoms can substitute for carbon atoms in the graphene lattice, creating defects and altering the material’s properties [16].

Doping with N or B disrupts the symmetry of the sp^2^ carbon network, leading to changes in charge distribution, the opening of a band gap, and shifting of the Fermi level. This enhances electron transfer and creates catalytically active sites [17,18]. It is reported that the possible introduction of N and B increases the number of defect sites and active centers in the graphene structure, which is beneficial for several applications like catalysis, sensing, and energy storage [19]. The effects of doping depend on the electronegativity and covalent radius of the dopant compared with carbon. N and B have different electronegativities and sizes, which leads to local structural changes and the creation of asymmetric spin density and active sites [20].

Metal oxides and doped materials have shown impressive performance due to recent experimental advancements in gas sensing. As an example, ceramics made of ZnGa_2_O_4_:Er have demonstrated effective methane sensing at high temperatures [21], whereas coatings based on ZnO display gas-sensitive luminescence behavior [22]. Furthermore, it has been reported that hexagonal boron nitride exhibits gas-dependent luminescence under different environmental conditions [23]. The significance of altering materials is underscored by these studies, which also furnish a robust experimental foundation for theoretical explorations of gas–surface interactions.

Recent DFT studies have shown that doping with heteroatoms can greatly enhance the sensing performance of graphene-based materials by altering their electronic structure and adsorption properties. Nonetheless, most documented studies have concentrated on either single dopants or specific analytes, leaving the synergistic effects of N/B co-doping on various gas molecules and humidity-related species inadequately understood [24,25].

Density Functional Theory (DFT) is widely used to model graphene doped with boron (B) and nitrogen (N), providing insights into electronic, thermal, and adsorption properties. DFT modeling of graphene oxide doped with N and B atoms reveals that the electronic, thermal, and adsorption properties can be finely tuned by controlling the type, concentration, and configuration of dopants. These insights are crucial for designing advanced materials for nanoelectronics, sensing, and catalysis [26,27,28].

DFT is a powerful, efficient, and widely used computational method that provides essential data for understanding and predicting the properties of a vast array of systems and molecules across multiple scientific disciplines [29,30,31]. It is reported that DFT is a vital computational method that provides essential data for many systems and molecules, especially when experimental data are limited or unavailable. Its predictive power, versatility, and efficiency make it indispensable for advancing research in various scientific fields [32,33].

Even though there has been significant advancement in sensors based on graphene oxide, our grasp of the synergistic effects of N/B co-doping is still lacking. The majority of earlier investigations have concentrated on pristine or singly doped systems, as well as specific target gases. The interplay of electronic structure modulation induced by co-doping, charge transfer, and adsorption behavior regarding NH_3_, CO_2_, and H_2_O remains incompletely understood. Consequently, the objective of this research is to offer a systematic theoretical examination of N/B co-doped graphene oxide and its potential use in gas sensing applications.

The aim of this work is to investigate the effect of nitrogen (N), boron (B), and co-doping (N/B) on the electronic structure and chemical reactivity of graphene oxide (GrO) using DFT calculations. This investigation is then to quantify the changes in global reactivity descriptors induced by doping. We correlate these descriptors with the predicted interaction strength and sensing performance of the doped and co-doped GrO surfaces toward the target gas molecules (NH_3_, H_2_O, and CO_2_), identify the optimal doping configuration that maximizes them, and then perform IR analyses for the studied structures.

## 2. Molecular Modeling Computations

### 2.1. Building Model Molecules

**Figure 1:**
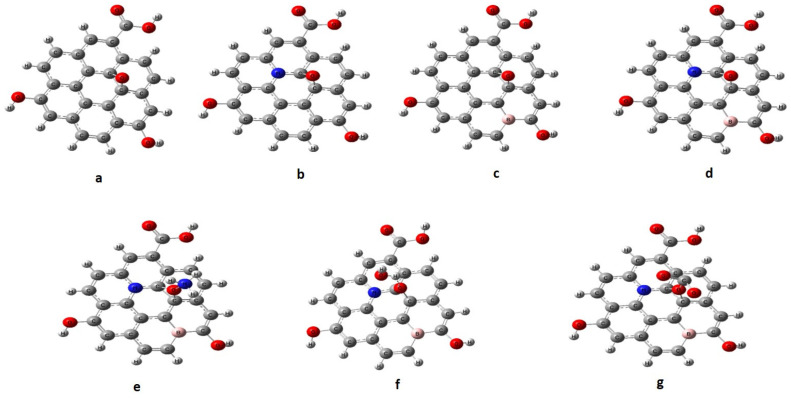
The studied model molecules, namely (**a**) graphene oxide (GrO), (**b**) graphene oxide doped with N (GrO-N), (**c**) graphene oxide doped with B (GrO-B), (**d**) graphene oxide doped with N and B (GrO-NB), (**e**) graphene oxide doped with N and B interacting with NH_3_ (GrO-NB-NH_3_), (**f**) graphene oxide doped with N and B interacting with H_2_O (GrO-NB-H_2_O), and (**g**) graphene oxide doped with N and B interacting with CO_2_ (GrO-NB-CO_2_).

The main model molecules of the study are represented by graphene oxide (GrO), which appears in Figure 1a. The GrO material undergoes nitrogen (N) and boron (B) interactions through a doping mechanism which results in heteroatoms substituting for carbon atoms within the graphene structure together with graphene oxide reduction. The method serves as a widely used approach to modify both the material structure and the electrical behavior for multiple usage scenarios. Nitrogen and boron atoms become part of the graphene structure through their substitution of carbon atoms. The process generally receives the name of heteroatom doping. Nitrogen has one more valence electron than carbon and, due to its higher electronegativity, it often acts as an electron donor or an n-type dopant. The model molecule in Figure 1b shows a graphene oxide structure which contains nitrogen dopants (GrO-N). Boron requires one additional valence electron compared with carbon, which enables it to function as an electron acceptor in the role of a p-type dopant. The model of molecules which represent graphene oxide with boron doping is shown in Figure 1c. The process enables N and B atoms to replace two carbon atoms through their incorporation into the graphene lattice. The last model is supposed to interact with three gases, namely NH_3_, H_2_O, and CO_2_. Figure 1e shows graphene oxide doped with N and B interacting with NH_3_ (GrO-NB-NH_3_). Figure 1f indicates graphene oxide doped with N and B interacting with H_2_O (GrO-NB-H_2_O). The final figure presents the model which displays graphene oxide with N and B doping that interacts with CO_2_ (GrO-NB-CO_2_).

### 2.2. Calculations Details

All the studied structures which mimic the interaction between nitrogen and boron with GrO were calculated with G09 program [34] installed at the Molecular Modeling and Spectroscopy Laboratory, Centre of Excellence for Advanced Science, National Research Centre, Egypt. Structures were optimized with B3LYP [35,36,37], namely Becke’s three-parameter hybrid functional with Lee–Yang–Parr correlation, which provides reliable results for a wide range of properties, and the triple-zeta valence split diffuse polarization function, 6-311G+(d,p). The choice of a functional and a basis set is a balance between accuracy, computational cost, and physical relevance. The combination of B3LYP and 6-311G+(d,p) is widely considered a “workhorse” in the field. It is sufficient to resolve most structural and energetic properties for medium-sized molecules. Therefore, B3LYP/6-311G+(d,p) is considered to be a relatively high-quality and common choice for obtaining accurate molecular properties.

Geometry optimizations were performed using the Berny optimization algorithm in Gaussian 09, applying standard convergence thresholds of 0.000450 a.u. for the maximum force, 0.000300 a.u. for the RMS force, 0.001800 a.u. for the maximum displacement, and 0.001200 a.u. for the RMS displacement. The Self-Consistent Field (SCF) procedure utilized a strict density matrix convergence threshold of 1.00 × 10^−8^ on the Root Mean Square (RMS) density matrix, corresponding to the default Gaussian energy convergence criterion of 1.00 × 10^−8^ a.u. on successive SCF iterations.

Total dipole moment TDM, HOMO/LUMO energy, and molecular electrostatic potential (MESP) were calculated, then frequency calculations were performed at the same level of theory. Global reactivity descriptors are essential computational tools for understanding and predicting the chemical behavior of molecules, calculated primarily from HOMO and LUMO energies using DFT methods, and include parameters such as ionization potential (IE), electronic affinity (A), chemical potential (μ), chemical hardness (η), absolute softness (S), and electrophilicity index (ω). These were computed with the following equations [38,39]:

(1) $$IP=-E_{HOMO}$$

(2) $$A=-E_{LUMO}$$

(3) $$\mu =\frac{IP+A}{2}$$

(4) $$\eta =\frac{IE-A}{2}$$

(5) $$S=\frac{1}{\eta }$$

(6) $$\omega =\frac{\mu^{2}}{2\eta }$$

To assess the stability of the studied models as well as the intermolecular interactions after hydration, the quantum theory of atoms in molecules (QTAIM) and non-covalent interactions (NCI) coupled with a reduced density gradient were conducted using Multiwfn and visual molecular dynamics (VMD) software [40,41]. The present calculations utilize a finite graphene oxide cluster model, which is commonly used to study local electronic and adsorption properties. Even though finite-size and edge effects may affect the absolute values, the calculated trends are still meaningful because all systems were treated within the same computational framework.

## 3. Results

### 3.1. TDM and HOMO/LUMO Energy Gap (ΔE)

The research team analyzed the total dipole moment and HOMO/LUMO energy gap (∆E) results, which they obtained from their study of the examined structures. The electronic characteristics of the investigated structures display observable differences which occur through the dopant’s (N and B) introduction according to the information shown in Table 1. The baseline measurement for undoped graphite oxide (GrO) reveals a total dipole moment (TDM) value of 4.2188 Debye and a HOMO/LUMO energy gap measurement of 2.9059 eV. The introduction of nitrogen or boron significantly alters these properties. For the N-doping process which uses GrO-N, the TDM measurement rises to 4.8850 Debye while the gap measurement decreases to 1.3622 eV, which indicates that the material now has greater polarity and more active chemical properties than GrO. B-doping (GrO-B), while having a lower TDM (2.7989 Debye) than GrO-N, shows a more pronounced reduction in the HOMO/LUMO gap, dropping sharply to 1.3388 eV. The electronic properties of co-doped structures, GrO-NB, show a notable reduction. The total dipole moment (TDM) of GrO-NB decreased to 1.2696 Debye, and its HOMO/LUMO energy (∆E) dropped to 1.9897 eV.

Table 1 also presents the total dipole moment and HOMO/LUMO energy (∆E) for the studied structures after interaction with NH_3_, H_2_O, and CO_2_. The GrO-NB-H_2_O exhibits the largest TDM (3.8228 Debye), which suggests that the interaction with H_2_O causes the most significant charge transfer or polarization on the GrO-NB structure, indicating the strongest interaction among the three gases.

The GrO-NB-CO_2_ has the smallest TDM (1.5002 Debye), implying the weakest charge rearrangement or the least polar interaction. The GrO-NB-NH_3_ has an intermediate TDM (1.9864 Debye), suggesting an interaction strength between H_2_O and CO_2_. The GrO-NB-H_2_O has the smallest ΔE (1.9565 eV), so it takes less energy to excite an electron from the HOMO to the LUMO, leading to higher chemical reactivity and greater potential change in electrical conductivity upon gas adsorption, making the structure a potentially highly sensitive chemical sensor for H_2_O. The NH_3_ gap measures 2.0504 eV, and the CO_2_ gap measures 1.9843 eV, which are both larger than the H_2_O gap. The GrO-NB structure shows greater stability after these two gases interact with it, which results in a smaller change in conductivity when compared with H_2_O interactions.

**Figure 2:**
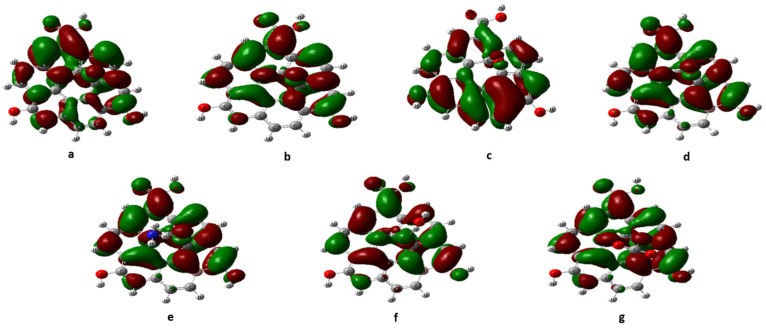
HOMO/LUMO for the studied model molecules: (**a**) GrO, (**b**) GrO-N, (**c**) GrO-B, (**d**) GrO-NB, (**e**) GrO-NB-NH_3_, (**f**) GrO-NB-H_2_O, and (**g**) GrO-NB-CO_2_.

The diagram in Figure 2 displays an examination of how different model structures based on graphene oxide (GrO) display their HOMO and LUMO spatial distributions, together with their corresponding energy levels. The graphene oxide (GrO) structure shown in Figure 2a functions as the reference control structure for its HOMO and LUMO elements. Figure 2b shows GrO modified with nitrogen (N), while Figure 2c shows GrO modified by boron (B) atoms’ introduction. Figure 2d shows graphene oxide doped with nitrogen and boron (GrO-NB).

Doping with N (GrO-N) creates p-type or n-type characteristics depending on the method of N atom integration into the GrO lattice. N acts as an n-type dopant (electron donor) because its valence electron count exceeds C, which leads to higher HOMO energy and lower LUMO energy, thus reducing the band gap (Eg) length. B (GrO-B) functions as a p-type dopant (electron acceptor) because it has fewer valence electrons than C, which causes reductions in HOMO energy and LUMO energy increases, thus producing potential band gap (Eg) decreases.

Co-doping (GrO-NB) achieves its desired results through disentangling the electronic band gap, which creates defects and charge redistribution that enhances charge separation. Graphene oxide doped with N and B interacts with NH_3_ and H_2_O, and CO_2_ gases, as demonstrated in Figure 2e–g.

H_2_O and other gases create an electron suction effect which draws electrons from the GrO-NB’s surface. The gas LUMO interacts with the GrO-NB HOMO according to the explanation. The GrO-NB component functions as an electron donor source. The GrO-NB surface receives electrons from NH_3_ and CO_2_ gas molecules. The gas HOMO interacts with the GrO-NB LUMO according to the explanation. The GrO-NB component functions as an electron acceptor source.

It should be emphasized that alterations in the HOMO–LUMO energy gap alone do not constitute a comprehensive measure of sensing performance. Consequently, the sensing behavior was evaluated through a combination of electronic analyses, reactivity analyses, and interaction analyses.

### 3.2. Computed Molecular Electrostatic Potential (MESP)

**Figure 3:**
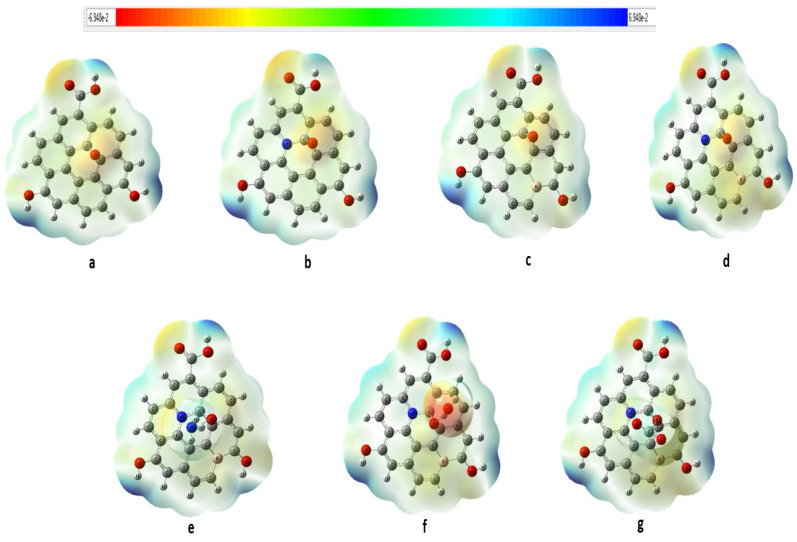
Mapped MESP for the studied model molecules: (**a**) GrO, (**b**) GrO-N, (**c**) GrO-B, (**d**) GrO-NB, (**e**) GrO-NB-NH_3_, (**f**) GrO-NB-H_2_O, and (**g**) GrO-NB-CO_2_.

The GrO map displays an uneven potential distribution which mainly results from the existence of oxygen-containing functional groups according to the information presented in Figure 3a. The strong red regions are localized around the highly electronegative oxygen atoms of the epoxy and hydroxyl groups. The blue regions are found near the hydrogen atoms of the O-H groups, indicating potential sites for hydrogen bonding. GrO serves as an intermediate electron donor/acceptor, while its reactivity occurs mainly through functional groups and the edges of the material.

The MESP map of GrO-N displays a higher negative potential through its more intense red regions compared with the GrO map, according to the information shown in Figure 3b. GrO-N becomes a better nucleophile because this process enhances its electron-donating capabilities. The structure will demonstrate better electrophilic interactions, which lead to increased chemical reactivity.

The MESP map of GrO-B shows positive potential in blue regions, which appear at positions that directly associate with the B atoms embedded within the base map (Figure 3c). The GrO-B material exhibits enhanced properties as an electrophile because of its stronger ability to accept electrons from other substances. The structure will demonstrate better nucleophilic interactions, which lead to increased chemical reactivity.

The nitrogen- and boron-doped graphene oxide material shows its presence through the results presented in Figure 3d. The GrO-NB MESP map displays its synergistic charge redistribution, which creates a highly active surface that shows multiple electron-rich (N) and electron-poor (B) sites that drive its enhanced chemical and electrical properties. The MESP regions on the surface of GrO-NB display an alternating pattern of positive (blue) and negative (red) areas because of the different total charge distribution, which results in better sensing capabilities due to their enhanced chemical and electrical characteristics. The three studied gases then interact with GrO-NB as indicated in Figure 3e,f,g, respectively.

The most striking feature is the dramatic change induced by H_2_O, as shown in Figure 3f. H_2_O acts as a strong oxidizing agent which typically oxidizes substances by accepting electrons from them. The resulting GrO-NB-H_2_O material exhibits an overall electron-rich character (red) which makes it highly vulnerable to further electrophilic attack or leads to a critical shift in the electrical conductivity of the graphene material.

The NH_3_ molecule functions as a base and an electron donor which produces mild localized effects according to the results shown in Figure 3e. The localized blue potential indicates that the N-H bonds become accessible for both hydrogen bonding and other types of nucleophilic interactions.

CO_2_ exhibits a poor interaction with pure graphene, as demonstrated by the results shown in Figure 3g. The MESP indicates that the N and B doping process creates a weak interaction between the two substances. The surface predominantly exhibits weak van der Waals forces. The surface exists in a condition between almost no electrical activity and a state of minimal positive electrical behavior.

### 3.3. Calculated IR Vibrational Spectra

GaussView 6.0. software was used to perform band assignment of the calculated IR spectra for the studied structures through visual inspection of the computed bands. The process requires showing animated normal modes for every calculated frequency, which scientists use to match those modes with their known chemical bond stretches and bends.

**Figure 4:**
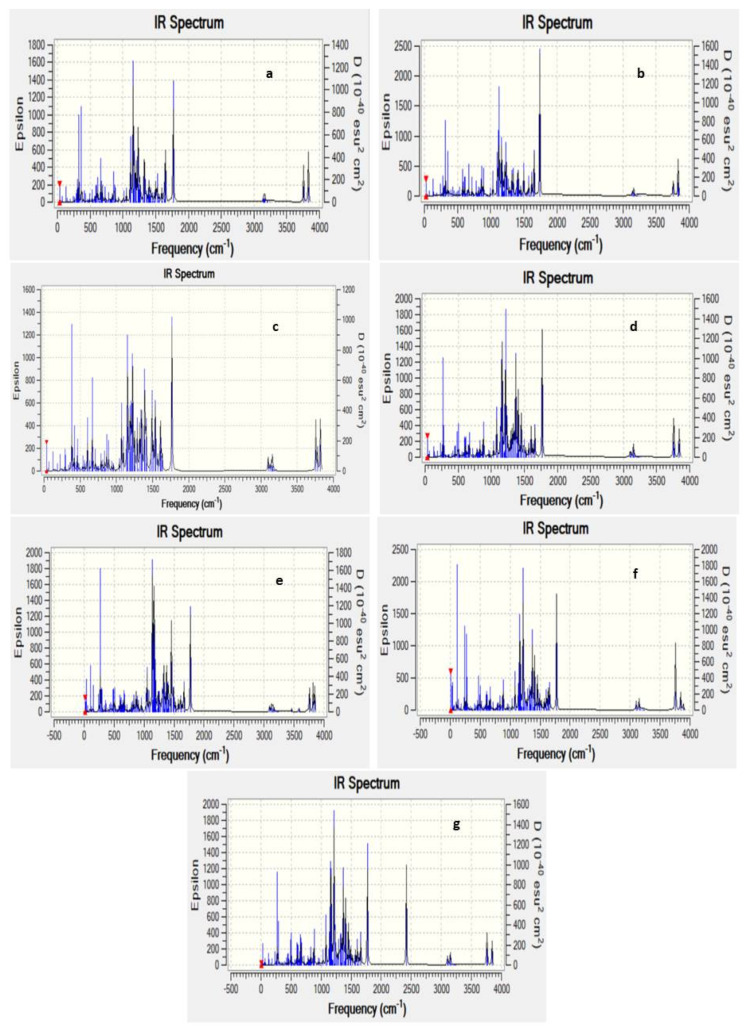
Calculated IR vibrations for the studied molecules: (**a**) GrO, (**b**) GrO-N, (**c**) GrO-B, (**d**) GrO-NB, (**e**) GrO-NB-NH_3_, (**f**) GrO-NB-H_2_O, and (**g**) GrO-NB-CO_2_.

The studied samples’ IR results are displayed in Figure 4. The calculated IR spectrum for GrO (Figure 4a) shows peaks that correspond to its main oxygen-containing functional groups, which include OH stretching (3400–3200 cm^−1^), C=O stretching (1750–1700 cm^−1^), C=C stretching (1600–1580 cm^−1^), and C-O stretching (1250–1000 cm^−1^).

The presence of N-H stretching vibrations, which would normally exist in the 3300–3500 cm^−1^ range, serves as confirmation that nitrogen atoms have been incorporated into the material, as shown in Figure 4b. The band overlaps with the OH band. The C-N stretching appears in the 1300–1350 cm^−1^ range. The C=C peak at 1600 cm^−1^ experiences both a potential shift and an increase in intensity because the electronegative N atom disrupts the π-electron system, which brings back some sp2 conjugation.

The process of confirming boron’s incorporation through IR becomes more difficult because B-C and B-O bonds produce vibrations that occur within the low-frequency fingerprint region which extends below 1300 cm^−1^.

The region at 1100–1250 cm^−1^ contains bands that scientists identify as B-O stretching (Figure 4c). B doping results in a new electronic environment which affects the carbon network that surrounds it. The calculated frequencies for C=O (carbonyl/carboxyl) and C-O (epoxy/alkoxy) stretching will undergo blue-shifts (higher frequencies) because the boron atom attracts electrons, which strengthens the nearby chemical bonds.

The GrO-NB spectra (Figure 4d,e) display the characteristics of both N and B doping, which result in intricate spectral overlaps and potential shifts that occur from N and B atoms’ interactions when they are near each other, as shown in Figure 4d. This study uses Table 2 to show the vibrational features of the research, which includes the band assignment for all studied structures. The study presents Table 3 to show the features induced by doping, including GrO-N, GrO-B, and GrO-NB.

### 3.4. Global Reactivity Descriptors

The global reactivity descriptors were calculated from the Highest Occupied Molecular Orbital energy (EHOMO) and the Lowest Unoccupied Molecular Orbital energy (ELUMO), and their results appear in Table 4. The calculated global reactivity descriptors demonstrate that doping causes major changes in the electronic characteristics of graphene oxide. GrO exhibits a chemical hardness of 1.4530 eV and a softness value of 0.6883 eV^−1^, together with its electrophilicity value of 5.4745 eV.

The nitrogen doping process in GrO-N results in a reduction in hardness to 1.0222 eV, while the softness value increases to 0.9783 eV^−1^, and the electrophilicity value decreases slightly to 5.3121 eV, which shows its capacity to donate electrons. GrO-B demonstrates higher hardness at 1.4697 eV, while its softness drops to 0.6804 eV^−1^, and its electrophilicity value remains at 5.4414 eV because it functions as an electron acceptor.

The GrO-NB co-doped system experienced a reduction in hardness to 0.9949 eV, while its softness increased to 1.0052 eV^−1^, and its electrophilicity experienced a major rise, which reached 8.8845 eV. Molecular adsorption enhances this effect because GrO-NB-H_2_O shows the highest electrophilicity value of 9.7566 eV and a hardness value of 0.9783 eV, while GrO-NB-CO_2_ has an electrophilicity of 9.0071 eV and a hardness of 0.9921 eV, and GrO-NB-NH_3_ shows 8.5742 eV of electrophilicity and 1.0252 eV of hardness. The results show that graphene oxide systems attain improved reactivity and sensing capability through the processes of co-doping and adsorption. Doping has a major impact on the electronic reactivity and charge transfer behavior of graphene oxide according to the findings. N doping enhances electrons’ mobility through its electron-donating properties, which leads to increased softness and decreased hardness. B doping keeps the hardness at a higher level because it uses its electron-accepting properties. The dual doping system (GrO-NB) shows a stronger enhancement effect which manifests through its lower hardness and greater softness, which indicate both increased chemical reactivity and enhanced external entity interaction. The electrophilicity of molecules rises when NH_3_, H_2_O, and CO_2_ are adsorbed, which indicates both strong adsorption affinity and increased charge transfer capacity. The sensing capabilities of graphene oxide become more sensitive and appropriate for applications when co-doping occurs with molecular adsorption.

### 3.5. Computed Density of States

**Figure 5:**
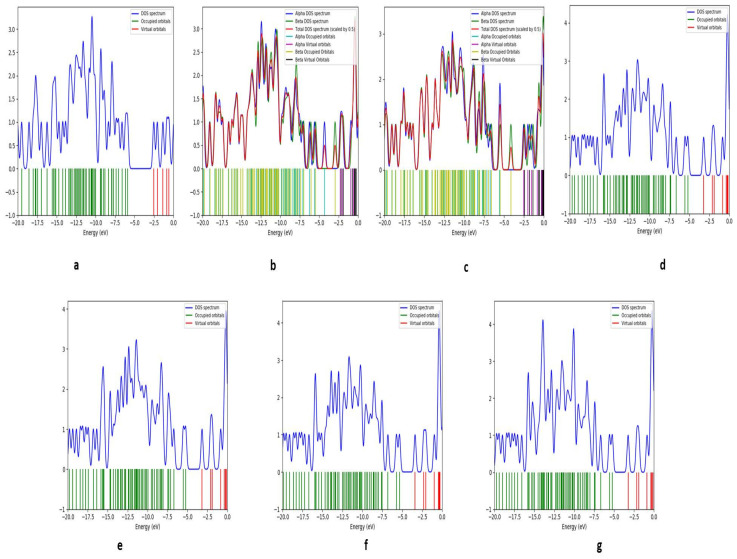
Calculated TDOS for the studied molecules: (**a**) GrO, (**b**) GrO-N, (**c**) GrO-B, (**d**) GrO-NB, (**e**) GrO-NB-NH_3_, (**f**) GrO-NB-H_2_O, and (**g**) GrO-NB-CO_2_.

The density of states was computed as the total density of states (TDOS), as shown in Figure 5, which provides complete insight into the electronic changes which occur during GrO’s functionalization and doping processes.

Based on G09 output files, both DOS and TDOS were generated using the Gauss Sum program [42]. The energy gap underwent significant changes when comparing unmodified GrO with systems that contained N and B and NB co-doping (Figure 5b–d), which resulted in new states appearing within the forbidden region. The GrO-NB configuration shows its most significant energy gap reduction because the N-2p and B-2p orbital hybridization with the carbon π-system results in better charge carrier mobility. The GrO-N and GrO-B plots (Figure 5b,c) display spin-polarized distributions which demonstrate electronic asymmetry between the alpha and beta channels that p-type or n-type doping creates through localized magnetic moments. The adsorption of NH_3_, H_2_O, and CO_2_ molecules (see Figure 5e–g) causes noticeable disturbances in the TDOS. The GrO-NB-NH_3_ complex shows marked displacement and expansion of its virtual (red) and occupied (green) orbital peaks (Figure 5e). The TDOS changes establish molecular interaction patterns which demonstrate that the NB-doped GrO surface achieves heightened sensitivity and selectivity.

### 3.6. Analysis of QTAIM Topology

The Quantum Theory of Atoms in Molecules (QTAIM) is a robust framework for analyzing electrons’ density distributions by identifying bond paths and critical points. By evaluating the topological properties at bond critical points (BCPs), one can characterize the nature and strength of interatomic connections [43,44,45].

**Figure 6:**
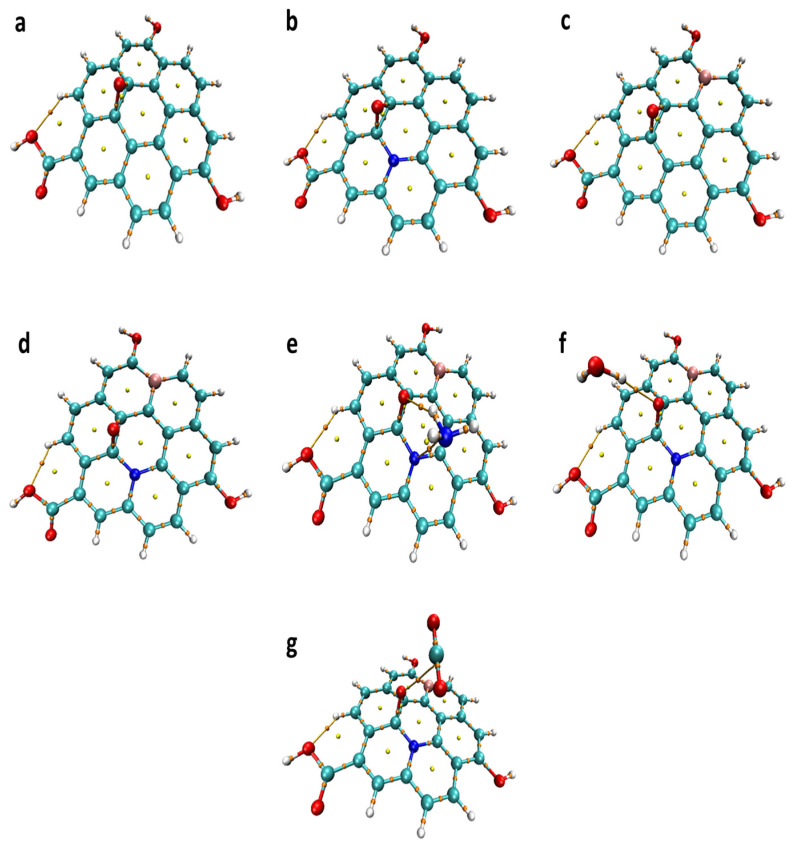
Computed QTAIM topology for the studied models: (**a**) GrO, (**b**) GrO-N, (**c**) GrO-B, (**d**) GrO-NB, (**e**) GrO-NB-NH_3_, (**f**) GrO-NB-H_2_O, and (**g**) GrO-NB-CO_2_.

Typically, stronger covalent interactions are characterized by a high electron density ρ(r), as well as negative values for both the Laplacian and the total energy density ρ(r). Conversely, non-covalent (closed-shell) interactions such as hydrogen bonding or van der Waals forces are identified by ∇^2^ρ(r) > 0 and H(r) > 0. The QTAIM topology for the investigated structures is illustrated in Figure 6a–g, displaying the identified bond paths and critical points.

The calculated topological parameters ρ(r), ∇^2^ρ(r), and H(r) indicate that the interactions between the oxygen atoms of GrO and the CO_2_, NH_3_, and H_2_O molecules are primarily van der Waals in nature. This is evidenced by the low electron density values at the BCPs and the positive values of both the Laplacian and total energy density, which are characteristic of weak, closed-shell interactions.

The interactions were classified as weak closed-shell interactions based on the QTAIM descriptors, specifically the low electron density at the bond critical points along with positive values of ∇^2^ρ(r) and H(r), which are indicative of van der Waals interactions.

### 3.7. Computed Non-Covalent Interaction (NCI) and Reduced Density Gradient (RDG)

**Figure 7:**
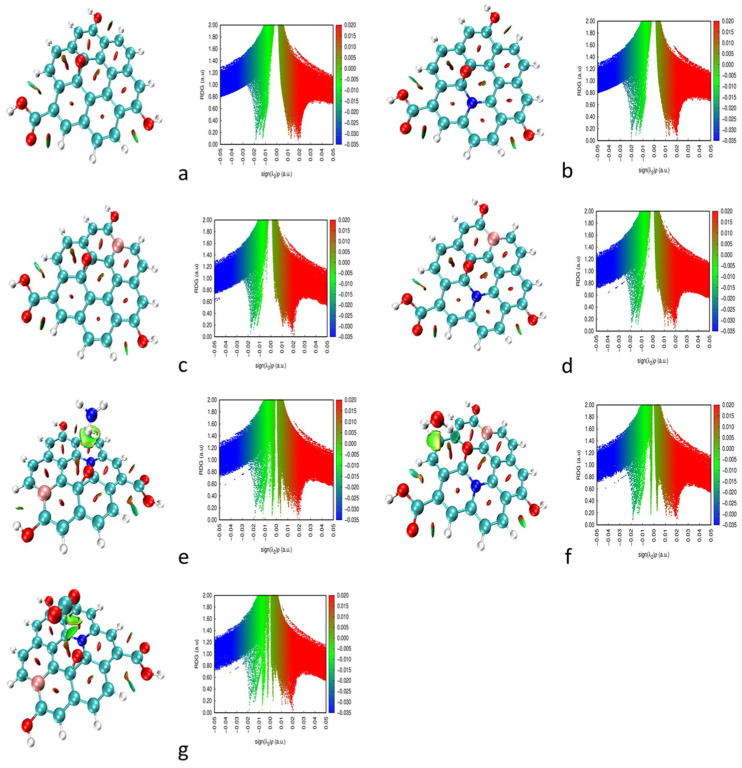
Computed NCI and RDG plots for studied structures: (**a**) GrO, (**b**) GrO-N, (**c**) GrO-B, (**d**) GrO-NB, (**e**) GrO-NB-NH_3_, (**f**) GrO-NB-H_2_O, and (**g**) GrO-NB-CO_2_.

To gain a deeper understanding of the intermolecular interactions within the composites, Non-Covalent Interaction (NCI) analysis and Reduced Density Gradient (RDG) mapping were used. These techniques visualize non-bonded interactions including hydrogen bonding, van der Waals forces, and steric repulsion via color-coded isosurfaces and gradient plots [46]. In the NCI-RDG plots (Figure 7), blue regions identify strong attractive interactions such as hydrogen bonds, green regions represent weaker dispersive van der Waals forces, and red regions indicate areas of significant steric repulsion.

The NCI isosurfaces for all gas-interaction systems (Figure 7e–g) reveal green regions distributed along the interface, which correspond to both hydrogen bonding and dispersive forces. Furthermore, the RDG plots for all systems exhibit distinct green spikes, confirming the presence of van der Waals interactions between the gases and the doped GrO. For GrO-NB, Figure 7g has greener isosurfaces and spikes in the RDG, suggesting more van der Waals interactions.

A reversible sensing mechanism is supported by the dominance of weak non-covalent interactions, which allows adsorption-induced electronic perturbations to take place without strong covalent bond formation, thus enhancing both sensitivity and recovery.

### 3.8. Adsorption Energy Calculation

The adsorption energy is a key factor in assessing how strongly gas molecules interact with the sensing surface. Thus, the adsorption energies of NH_3_, CO_2_, and H_2_O on graphene oxide in its pristine form, as well as with N-doping, B-doping, and N/B-co-doping, were computed. The following equation [47,48] was used to calculate the adsorption energy, which is the difference between the total energy of the adsorbate–surface complex and the energies of the isolated gas molecule and surface:

E_Ads_ = [ESystem − (EA_dsorbent_ + E_Adsorbate_)]

To assess the sensing performance of the N/B-co-doped graphene oxide (GrO-NB) system, the adsorption energies of NH_3_, CO_2_, and H_2_O molecules were calculated (Table 5). The calculated adsorption energies are negative and range from −0.0735 to −0.1986 eV, which indicates that all adsorption processes are thermodynamically favorable and exothermic. Among the gases examined, NH_3_ demonstrates the most robust interaction with the GrO-NB surface (−0.1986 eV), succeeded by H_2_O (−0.1742 eV). In contrast, CO_2_ displays the weakest adsorption (−0.0735 eV), indicative of varying adsorption affinities and implying a potential selectivity for NH_3_.

The computed adsorption energies are in the physisorption range, validating a reversible adsorption behavior that benefits gas sensing applications. These results align with the electronic structure, DOS, and QTAIM/NCI analyses, further reinforcing the appropriateness of N/B-co-doped graphene oxide as a promising gas sensing material.

The present results are consistent with previous DFT studies that found enhanced adsorption and charge transfer interactions in doped graphene-based sensors for NH_3_, CO_2_, and H_2_O molecules. Nonetheless, when compared with pristine and singly doped graphene systems, N/B co-doped graphene oxide exhibits a more pronounced alteration of its electronic structure and enhanced interactions that depend on the analyte, indicating a synergistic effect of dual doping on the sensing performance. The findings correspond with recent theoretical investigations demonstrating that co-doping with heteroatoms can improve the sensitivity and selectivity of graphene-based sensing materials [49,50,51].

## 4. Conclusions

The study demonstrates, through Density Functional Theory (DFT) research, that nitrogen (N) and boron (B) doping enables precise control over the electronic and chemical characteristics of graphene oxide (GrO).

The research leads to several key conclusions regarding the material’s reactivity and its effectiveness as a gas sensor.

The introduction of N or B atoms into the GrO lattice leads to a substantial reduction in the HOMO/LUMO energy gap (ΔE), which results in increased chemical reactivity together with improved polarity of the material. The co-doping process which involves both N and B elements results in GrO-NB, producing a stable surface that offers high customization options through its electronic band gap measurement of 1.9897 eV. The GrO-NB surface demonstrates its highest interaction potential with H_2_O molecules among the tested gases, which include NH_3_, H_2_O, and CO_2_. The total dipole moment reaches its maximum value at 3.8228 Debye, while the energy gap attains its minimum value of 1.9565 eV, which demonstrates significant charge transfer and heightened sensitivity to water vapor.

Compared with pristine and singly doped structures, the co-doped GrO-NB system demonstrates a stable configuration and enhanced electronic properties. Calculations of the adsorption energy show that interactions with NH_3_, H_2_O, and CO_2_ molecules are favorable and reversible, with values of −0.1986 eV, −0.1742 eV, and −0.0735 eV, respectively. The values confirm that physisorption dominates the adsorption process, making it suitable for gas sensing applications due to the balance between sensitivity and reversibility.

The study’s findings have the potential to create applications across both nanotechnology and environmental monitoring. The GrO-NB structure enables the development of advanced chemical sensors which detect H_2_O vapor through the ability to deliver high sensitivity based on its drastic conductivity alterations during adsorption. The ability to “fine-tune” the band gap through substitutional doping makes N/B co-doped graphene oxide a prime candidate for designing specialized components in nanoelectronic devices.

The findings highlight the potential of N/B-co-doped graphene oxide for gas sensing applications and provide valuable insights into the relationships among co-doping, electronic structure modulation, and gas adsorption behavior.

## Figures and Tables

**Table 1:** Computed total dipole moment (TDM) as Debye and HOMO/LUMO energy gap (ΔE) as eV for the studied model molecules.

Structure	TDM (Debye)	∆E (eV)
GrO	4.2188	2.9059
GrON	4.8850	1.3622
GrO-B	2.7989	1.3388
GrO-NB	1.2696	1.9897
GrO-NB-NH_3_	1.9864	2.0504
GrO-NB-H_2_O	3.8228	1.9565
GrO-NB-CO_2_	1.5002	1.9843

**Table 2:** Band assignment for the studied structures.

Band Position (cm^−1^)	Assignment	Vibration Type	
∼3400−3200	ν(O-H)	Stretching	GrO (a), GrO-N (b), GrO-B (c), GrO-NB (d, e)
∼1750−1700	ν(C=O)	Stretching	GrO, GrO-N, GrO-B, GrO-NB (carboxyl/carbonyl)
∼1600−1560	ν(C=C)	Stretching	GrO, GrO-N, GrO-B, GrO-NB (aromatic/skeletal)
∼1250−1180	ν(C-O-C)	Stretching	GrO, GrO-N, GrO-B, GrO-NB (epoxy/ether)
∼1050−1000	ν(C-O)	Stretching	GrO, GrO-N, GrO-B, GrO-NB (alkoxy/hydroxyl)

**Table 3:** Doping-induced features (GrO-N, GrO-B, GrO-NB).

Doping Type	Wavenumber Range (cm^−1^)	Assignment	
Nitrogen (N)	1500∼450	ν(C=N)	C=N stretching from pyridinic or graphitic N structures.
(b, d, e)	1350∼1180	ν(C-N)	C-N stretching from pyrrolic or amine structures. This band often overlaps with C-O bands.
Boron (B)	1400∼1300	ν(B-C)	B substitution in the carbon lattice (graphitic B).
(c, d, e)	1200∼1000	ν(B-O) or ν(B-C2O)	Vibrations associated with B bonded to oxygen or C atoms near oxygenated regions (boronic acid/ester-like structures).
GrO-NB	1200∼1500	ν(C-N, C-B, C-O)	Co-doping (d, e) shows complex peak shifting and broadening in this region due to electronic interactions between adjacent N and B atoms.

**Table 4:** Computed global reactivity descriptors for the studied model molecules.

Model Molecule	I (eV)	A (eV)	μ (eV)	η (eV)	S (eV)^−1^	ω (eV)
GrO	5.4415	2.5356	3.9885	1.4530	0.6883	5.4745
GrO-N	4.3177	2.2733	3.2955	1.0222	0.9783	5.3121
GrO-B	5.4690	2.5296	3.9993	1.4697	0.6804	5.4414
GrO-NB	5.1993	3.2096	4.2045	0.9949	1.0052	8.8845
GrO-NB-NH_3_	5.2181	3.1677	4.1929	1.0252	0.9754	8.5742
GrO-NB-H_2_O	5.3474	3.3909	4.3691	0.9783	1.0222	9.7566
GrO-NB-CO_2_	5.2198	3.2355	4.2276	0.9921	1.0079	9.0071

**Table 5:** Calculated total energy and adsorption energy for the studied structures of GrO-NB and those interacting with NH_3_, H_2_O, and CO_2_.

Structure	Total Energy (a.u.)	Adsorption Energy (a.u.)	Adsorption Energy (eV)
NH_3_	−56.5826		
H_2_O	−76.4585		
CO_2_	−188.6469		
GrO-NB	−1339.7332		
GrO-NB-NH_3_	−1396.3231	−0.0073	−0.1986
GrO-NB-H_2_O	−1416.1981	−0.0064	−0.1742
GrO-NB-CO_2_	−1528.3828	−0.0027	−0.0735

## Data Availability

The original contributions presented in this study are included in the article. Further inquiries can be directed to the corresponding authors.

## References

[1] Sharma A., Sharma S., Yadav S., Arora B., Dutta S., Dixit R., Mehta S., Sharma R.K. (2024). Covalently functionalized graphene oxide metal complexes: Versatile nanocatalysts for organic transformations. Mater. Sci. Eng. B.

[2] Elsayed E., Wang H., Anderson P.A., Al-Dadah R., Mahmoud S., Navarro H., Ding Y., Bowen J. (2017). Development of MIL-101(Cr)/GrO composites for adsorption heat pump applications. Microporous Mesoporous Mater..

[3] Borane N., Boddula R., Odedara N., Singh J., Andhe M., Patel R. (2024). Comprehensive review on synthetic methods and functionalization of graphene oxide: Emerging applications. Nano-Struct. Nano-Objects.

[4] Veeresh S., Ganesha H., Nagaraju Y., Vijeth H., Vandana M., Basappa M., Devendrappa H. (2022). Graphene oxide/cobalt oxide nanocomposite for high-performance electrode for supercapacitor application. J. Energy Storage.

[5] Iranshahi S., Mosivand S. (2022). Cobalt/graphene oxide nanocomposites: Electro-synthesis, structural, magnetic, and electrical properties. Ceram. Int..

[6] Bashiri M., Hosseini-Sarvari M. (2024). A comprehensive investigation into the synthesis, characterization, and photocatalytic performance of modified graphene oxide via imino bond with ferrocene as a novel photocatalyst for thioamide synthesis. Catal. Sci. Technol..

[7] Chou S., Lin S., Wu Y., Huang T., Lin M., Lee Y., Weng S., Chen H., Wu Y. (2025). A novel method for synthesis of graphene oxide thin-film utilizing vacuum UV exposure. Opt. Mater..

[8] Moutcine A., Laghlimi C., Ziat Y., Bahraoui S.E., Belkhanchi H., Jouaiti A. (2024). Advanced design of chemically modified electrodes for the electrochemical analysis of uric acid and xanthine. J. Pharm. Biomed. Anal..

[9] Liu Y., Ma Y., Yang J., Zhang S., Wu N., Wang P., Wang L. (2025). Overcoming chemical stability challenges of nanoporous graphene separation membranes in harsh environments. Chem. Eng. J..

[10] Krivanek O.L., Chisholm M.F., Murfitt M.F., Dellby N. (2012). Scanning transmission electron microscopy: Albert Crewe’s vision and beyond. Ultramicroscopy.

[11] Lan N.T., Chi D.T., Dinh N.X., Hung N.D., Lan H., Tuan P.A., Thang L.H., Trung N.N., Hoa N.Q., Huy T.Q. (2014). Photochemical decoration of silver nanoparticles on graphene oxide nanosheets and their optical characterization. J. Alloys Compd..

[12] Stefan-Henningsen E., Roberts N., Kiani A. (2025). Enhancing tribological performance: A comprehensive review of graphene-based additives in lubricating greases. Results Eng..

[13] Xue X., Li S., Zhu M. (2025). Recent progress in graphene-based materials for thermoelectric applications. RSC Adv..

[14] Wijewardena L.H.A., Cheon W.S., Jeong S., Park J., Jang H.W. (2025). Graphene-based catalysts for electrochemical CO_2_ reduction reaction. RSC Sustain..

[15] Qi T., Xiong K., Zhang X. (2024). Research progress of carbon materials in the anodes of sodium-ion batteries. J. Power Sources.

[16] Chen G., Yuan B., Wang Y., Chen X., Huang C., Shang S., Tao H., Liu J., Sun W., Yang P. (2020). Nacre-biomimetic graphene oxide paper intercalated by phytic acid and its ultrafast fire-alarm application. J. Colloid Interface Sci..

[17] Guo Z., Feng Y., Chen Y., Yao Q., Luo H., Chen X. (2019). A taurine-functionalized 3D graphene-based foam for electrochemical determination of hydrogen peroxide. Talanta.

[18] Sun Y., Du C., Han G., Qu Y., Du L., Wang Y., Chen G., Gao Y., Yin G. (2016). Boron, nitrogen co-doped graphene: A superior electrocatalyst support and enhancing mechanism for methanol electrooxidation. Electrochim. Acta.

[19] Pastrana-Martínez L.M., Morales-Torres S., Figueiredo J.L., Faria J.L., Silva A.M. (2018). Graphene photocatalysts. Multifunctional Photocatalytic Materials for Energy.

[20] Walke P.S., Gupta S.P., Nishad H., Sathe B.R., Late D.J. (2021). Engineering two-dimensional materials for high-performance supercapacitor devices. Fundamentals and Supercapacitor Applications of 2D Materials.

[21] Almaev A.V., Karipbayev Z.T., Kakimov A.B., Yakovlev N.N., Kukenov O.I., Korchemagin A.O., Akmetova-Abdik G.A., Kumarbekov K.K., Zhunusbekov A.M., Mochalov L.A. (2025). High-Temperature Methane Sensors Based on ZnGa_2_O_4_: Er Ceramics for Combustion Monitoring. Technologies.

[22] Grigorjeva L., Millers D., Smits K., Zolotarjovs A. (2015). Gas sensitive luminescence of ZnO coatings obtained by plazma electrolytic oxidation. Sens. Actuators A Phys..

[23] Korsaks V. (2015). Hexagonal boron nitride luminescence dependent on vacuum level and surrounding gases. Mater. Res. Bull..

[24] Karunarathne S., Malaarachchi C.K., Abdelkader A.M., Kamali A.R. (2024). Advances in bifunctional electrocatalysts towards high-performing Li-air batteries. J. Power Sources.

[25] Patil U., Mahakal S., Nerkar D., Noothalapati H., Shinde S., Waghmode S.A., Amalnerkar D. (2025). Nanoarchitectured heteroatom-doped graphene as an adsorbent for dyes and heavy metals: Historical account, recent advancement and future prospects. Inorg. Chem. Commun..

[26] Abdullah N.R., Abdullah B.J., Tang C., Gudmundsson V. (2021). Properties of BC_6_N monolayer derived by first-principle computation: Influences of interactions between dopant atoms on thermoelectric and optical properties. Mater. Sci. Semicond. Process..

[27] Tiwari A., Palepu J., Choudhury A., Bhattacharya S., Kanungo S. (2022). Theoretical analysis of the NH_3_, NO, and NO_2_ adsorption on boron-nitrogen and boron-phosphorous co-doped monolayer graphene—A comparative study. FlatChem.

[28] Todkar R., Shirote P., Mohite S. (2025). In Silico Screening and DFT Analysis of *Nelumbo nucifera* Phytochemicals as Potential BACE-1 Inhibitors for Alzheimer’s disease. Prospect. Pharm. Sci..

[29] Ibrahim A., Elhaes H., Khaled N.A., Ibrahim M.A. (2025). On the analyses of graphene oxide/polypyrrole/zinc oxide nanocomposites. Sci. Rep..

[30] El-Sheikh A.S., Abdelaziz N.S., Amin K.S., Elhaes H., Ibrahim M.A. (2025). Application of chitosan/graphene and chitosan/graphene oxide composites for removal of Cu and Pb. Sci. Rep..

[31] Ibrahim M. (2009). Molecular modeling and FTIR study for K, Na, Ca and Mg coordination with organic acid. J. Comput. Theor. Nanosci..

[32] Munir Z., Rehman A., Abbasi M.A., Siddiqui S.Z., Iqbal J., Amjad H., Hamid S., Momen A., Khalid H., Rasool S. (2025). Synthesis, biological analysis and computational studies of substituted 1,2,4-triazoles: FMO, MEP and docking investigations. J. Mol. Struct..

[33] Yalazan H., Koç D., Kose F.A., Akgül M.İ., Fandaklı S., Tüzün B., Taslimi P., Kantekin H. (2025). Chalcone-based Schiff bases: Design, synthesis, structural characterization and biological effects. J. Mol. Struct..

[34] Frisch M.J., Trucks G.W., Schlegel J., Scuseria G.E., Robb M.A., Cheeseman J.R., Schlegel H.B., Scalmani G., Barone V., Mennucci B. (2010). Gaussian 09.

[35] Becke A.D. (1992). Density-functional thermochemistry. I. The effect of the exchange-only gradient correction. J. Chem. Phys..

[36] Petersson G.A., Al-Laham M.A. (1991). A complete basis set model chemistry. II. Open-shell systems and the total energies of the first-row atoms. J. Chem. Phys..

[37] Lee C., Yang W., Parr R.G. (1988). Development of the Colle-Salvetti correlation-energy formula into a functional of the electron density. Phys. Rev. B.

[38] Ramírez-Martínez C., Zárate-Hernández L.A., Camacho-Mendoza R.L., González-Montiel S., Meneses-Viveros A., Cruz-Borbolla J. (2023). The use of global and local reactivity descriptors of conceptual DFT to describe toxicity of benzoic acid derivatives. Comput. Theor. Chem..

[39] El-Sayed N.M., Elhaes H., Ibrahim A., Ibrahim M.A. (2024). Investigating the electronic properties of edge glycine/biopolymer/graphene quantum dots. Sci. Rep..

[40] Lu T., Chen F. (2012). Multiwfn: A multifunctional wavefunction analyzer. J. Comput. Chem..

[41] Humphrey W., Dalke A., Schulten K. (1996). VMD: Visual molecular dynamics. J. Mol. Graph..

[42] O’Boyle N.M., Tenderholt A.L., Langner K.M. (2008). cclib: A library for meaningful analysis of data from chemical computation. J. Comput. Chem..

[43] Bader R.F.W. (1998). A bond path: A universal indicator of bonded interactions. J. Phys. Chem. A.

[44] Bader R.F.W. (1991). A quantum theory of molecular structure and its applications. Chem. Rev..

[45] Bader R.F.W. (1990). Atoms in Molecules: A Quantum Theory.

[46] Smati S., Djafri A., Menad K., Boukabcha N., Rahmani R., Goudjil M., Djafri A. (2024). Synthesis, molecular structure, Hirshfeld surface analysis, NCI-RDG, spectral characterization analysis, nonlinear optical properties, and in silico molecular docking of (E)-3-(3-(2-methoxyphenyl)-4-methylthiazol-2(3H)-ylidene)benzo [4,5]imidazo [1,2-c]thiazole-1(3H)-thione. J. Mol. Struct..

[47] Yong Y., Cui H., Zhou Q., Su X., Kuang Y., Li X. (2019). C_2_N monolayer as NH_3_ and NO sensors: A DFT study. Appl. Surf. Sci..

[48] Li Z., Artrith N., Medford A.J., Nørskov J.K. (2023). AdsorbML: A leap in efficiency for adsorption energy calculations with machine learning. npj Comput. Mater..

[49] Peyghan A.A., Rastegar S.F., Hadipour N.L. (2014). DFT study of NH_3_ adsorption on pristine, Ni-and Si-doped graphynes. Phys. Lett. A.

[50] Zhang C.P., Li B., Shao Z.G. (2019). First-principle investigation of CO and CO_2_ adsorption on Fe-doped penta-graphene. Appl. Surf. Sci..

[51] Lin X., Ni J., Fang C. (2013). Adsorption capacity of H_2_O, NH_3_, CO, and NO_2_ on the pristine graphene. J. Appl. Phys..

